# Supplementary material for: Validation of the Greek Cardiovascular Diet Questionnaire 2 (CDQ-2) and Single-Center Cross-Sectional Insights into the Dietary Habits of Cardiovascular Patients
Source: Nutrients. 2025 May 12;17(10):1649. doi: 10.3390/nu17101649 (PMC12114533; doi:10.3390/nu17101649)
Supplement: Supplementary file 1 [file nutrients-17-01649-s001.zip › nutrients-3620856-supplementary.pdf]

CDQ-2 (in English)

1. **Do you eat cheese ? (1 serving = 30 g)**
   - ☐ a) less than 1 serving per day
   - ☐ b) 1 serving per day
   - ☐ c) 2 servings per day
   - ☐ d) 3 servings per day or more

2. **Do you eat dairy ?**
   (1 dairy = 1 yogurt or a glass of milk (15 cl) or milk-based dessert)
   - ☐ a) I don’t eat dairy or less than once a day
   - ☐ b) I eat fat free dairy or reduced fat dairy
   - ☐ c) I eat dairy once or twice per day
   - ☐ d) I eat dairy three times a day
   - ☐ e) I eat dairy four times a day or more

3. **Do you eat some meat (except poultry)?**
   that means pork, beef, veal, sheep or lamb:
   - ☐ a) None or once a week
   - ☐ b) 2 or 3 times a week
   - ☐ c) 4 or 6 times a week
   - ☐ d) once a day or more

4. **Do you eat fresh fish, frozen fish or canned fish (tuna, mackerel, sardine, herring) ?**
   - ☐ a) less than once a week
   - ☐ b) once a week
   - ☐ c) twice a week
   - ☐ d) three times a week
   - ☐ e) four times a week or more

5. **Do you eat cooked pork meats (except lean ham)**
   that means pâté, corned beef, bacon, pastrami, sausage…
   - ☐ a) 0 or 1 a week
   - ☐ b) twice or 3 times a week
   - ☐ c) 4 to 6 times a week
   - ☐ d) once a day
   - ☐ e) more than once a day

6. **Do you eat quiches, salted pies, pizzas, hamburgers or sandwiches with butter?**
   - ☐ a) 0 or 1 a week
   - ☐ b) twice or 3 times a week
   - ☐ c) 4 or 6 times a week
   - ☐ d) once a day or more

7. **Do you eat pastries or cakes or biscuits ?**
   - ☐ a) 0 or 1 piece a week
   - ☐ b) 2 or 4 pieces a week
   - ☐ c) 5 pieces a week or more

8. **Do you eat croissants or brioches**
   - ☐ a) 0 or 1 a week
   - ☐ b) 2 or 3 a week
   - ☐ c) 4 or 6 a week
   - ☐ d) once a day
   - ☐ e) more than once a day

9. Do you eat fruits ?
(1 serving = an average fruit for example 1 apple or 2 clementines …)

- ☐ a) Never or rarely
- ☐ b) 1 to 2 servings a week
- ☐ c) 3 to 6 servings a week
- ☐ d) 7 to 13 servings a week (more than 1 fruit a day)
- ☐ e) 14 servings a week or more (more than 2 fruits a day)

10. Do you drink fruit juice every day ? (pure juice, without added sugar)

- ☐a) no
- ☐b) yes, a glass a day
- ☐c) yes, 2 glasses a day or more

11. Do you eat cooked vegetables or vegetables soups (1 serving = a plate or a bol)

- ☐ a) Never or rarely
- ☐ b) 1 or 2 serving a week
- ☐ c) 3 to 6 serving a week (less than one serving a day)
- ☐ d) 1 serving a day
- ☐ e) more than one serving a day

12. Do you eat raw vegetables or salads ?

- ☐ a) Never or rarely
- ☐ b) 1 or 2 servings a week
- ☐ c) 3 to 6 servings a week (less than one serving a day)
- ☐ d) 1 serving a day
- ☐ e) more than one serving a day

13. Do you usually eat a margarine with a high omega 3 content ?

- ☐ a) no
- ☐ b) yes, at 1 meal a day
- ☐ c) yes, at 2 meals a day
- ☐ d) yes, at 3 meals a day or more

14. Do you eat butter on your toasts or in your dishes (one serving = 10 g)

- ☐ a) never or rarely
- ☐ b) 1 serving a day
- ☐ c) 2 servings a day or more

15. Do you cook with butter ?

- ☐ a) no
- ☐ b) yes, at 1 meal a day
- ☐ c) yes, at 2 meals a day

16. Do you usually use one of these oils : canola, soybean, nut ?

- ☐ a) no
- ☐ b) yes, less than 1 tablespoon a day
- ☐ c) yes, less than 2 tablespoons a day
- ☐ d) yes, 2 tablespoons a day or more

17. Do you usually use olive oil ?

- ☐ a) no
- ☐ b) yes, less than 1 tablespoon a day
- ☐ c) yes, 1 tablespoon a day
- ☐ d) yes, 2 tablespoons a day
- ☐ e) yes, 3 tablespoons a day
- ☐ f) yes, 4 tablespoons a day or more
